# Pulmonary Artery Intimal Sarcoma: A Diagnostic Challenge Using a Multimodal Approach

**DOI:** 10.1155/2020/6795272

**Published:** 2020-10-03

**Authors:** Rima Nakrani, Ho-Man Yeung, Jin Sun Kim, Bhishak Kamat, Maruti Kumaran

**Affiliations:** ^1^Department of Medicine, Temple University Hospital, Philadelphia, PA, USA; ^2^Department of Radiology, Temple University Hospital, Philadelphia, PA, USA

## Abstract

Pulmonary artery intimal sarcoma (PAIS) is a rare tumor without clear syndromic presentation other than nonspecific symptoms of cough, dyspnea, and weight loss. This diagnosis is difficult due to challenging radiographic interpretations of multiple imaging modalities. We present a case of a 60-year-old male, who presented to his pulmonologist and underwent a CT chest with IV contrast that initially suggested primary lung carcinoma. CT angiogram showed significant vascular filling defects suspicious of an intravascular mass, rather than vascular invasion by lung lesions. The PET/CT scans further suggested a malignant process, but indistinguishable between an extravascular or intravascular etiology. Taking these results together, they suggested an intravascular malignancy, prompting a tissue biopsy, which ultimately led to a diagnosis of PAIS with metastases. Establishing a definitive diagnosis is essential as treatment and prognosis are different for sarcoma compared to carcinoma. There is no standard treatment to date, and management often includes a multidisciplinary approach involving surgery, radiation, chemotherapy, and targeted therapy. PAIS is a rare entity that cannot be diagnosed clinically and needs a multimodality approach for its diagnosis.

## 1. Introduction

Pulmonary artery intimal sarcoma (PAIS) is a rare tumor with poor prognosis that mimics conditions such as pulmonary artery pseudoaneurysm, extravascular tumor invasion, and thromboembolism. Often, there are diagnostic delays as symptoms are nonspecific and diagnostic investigations can be time consuming. PAIS is a rare entity, and our case demonstrates the extensive clinical and radiographical investigations necessary to establish this diagnosis.

## 2. Case Presentation

A 60-year-old African American male presented to his pulmonologist with a one-month history of persistent productive cough. He has been producing clear mucous, without any blood. His cough has been progressively worse throughout the past month. His cough was worse during the day and exacerbated by cold beverages and cold air. The patient denied other symptoms including shortness of breath, dyspnea on exertion, chest pain, hemoptysis, subjective fevers, chills, or night sweats. He denied typical symptoms for seasonal allergies and gastroesophageal reflux. The patient has a past medical history of sickle cell trait and atrial fibrillation. His past surgical history is notable for a laparoscopic sleeve gastrectomy resulting in a 150-pound weight loss five years ago. He has a family history of sickle cell disease, diabetes, and peripheral vascular disease, without any cancer. He also denied any weight loss, changes in appetite, dysphagia, or odynophagia. He denies any history of tobacco use or other recreational drugs but has a previous history of alcohol use. The patient has no occupational exposure to hazardous material and is a bus operator for the past several years. He denied any recent travel. He lives alone and is functional and independent at home without any need for supplemental oxygen. The patient had experienced a similar cough approximately one year prior with associated fevers, otalgia, and chest congestion. At that time, he was treated with a course of antibiotics resulting in complete resolution of his symptoms.

**Figure 1:**
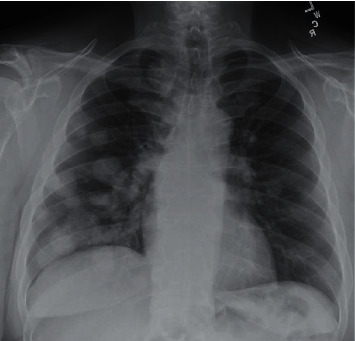
Initial chest X-ray showing multiple new pulmonary nodules.

**Figure 2:**
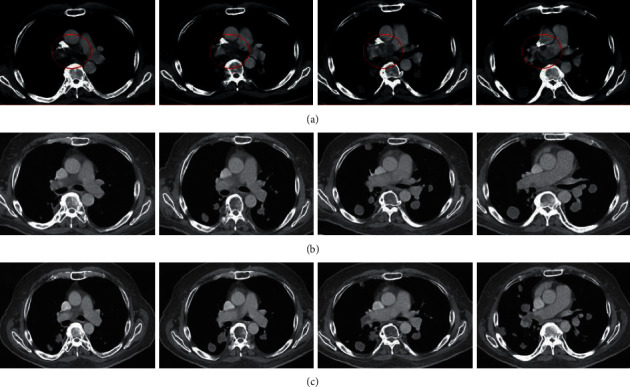
(a) Initial CT of the chest showing large intravascular mass encasing the right main pulmonary artery. (b) Repeat CT chest after 6 cycles of doxorubicin, displaying partial reduction of the primary pulmonary mass and metastatic lesions. (c) After 3 cycles of palbociclib, the patient had stable disease.

His vital signs on presentation were within normal limits, and he was saturating well above 95% on room air. On physical examination, the patient appeared comfortable and was in no respiratory distress. He was well developed and well nourished. He was alert and oriented to person, place, time, and events. Cardiovascular exam was unremarkable. Pulmonary exam revealed normal respiratory effort and normal breath sounds, with no stridor, wheezes, rhonchi, or rales. The patient also did not exhibit any palpable cervical, supraclavicular, axillary, or inguinal lymphadenopathy. Laboratory testing, including complete blood count and basic metabolic panel, did not reveal any abnormalities. A chest X-ray (Figure 1), in comparison to one year prior, revealed multiple new lung nodules. A chest CT with contrast demonstrated mediastinal adenopathy and multiple, large, round lung nodules with sharp borders predominantly in the right lung (a 3.3 cm nodule in the medial right upper lobe and two nodules in the right lower lobe, 4.8 cm and 3 cm, respectively) with mass effect on the right pulmonary artery and encasement of the right pulmonary artery, in addition to a left lower lobe nodule (2.2 cm) (Figure 2(a)).

Given the new radiographic findings, he was sent to the hospital for additional workup and was scheduled for CT-guided transthoracic needle biopsy. Tumor markers (alpha fetoprotein, B-HCG/LDH, CEA, and CA 19-9) at this time were all within the normal range. To further evaluate the patency of the pulmonary arteries, a chest CT angiogram was performed, which revealed a soft-tissue density mass in the right pulmonary artery with scattered bilateral lung parenchymal lesions concerning for metastatic pulmonary angiosarcoma with known osseous metastatic lesion involving T3 vertebrae. Fine-needle aspiration biopsy of the right lower lung mass was nondiagnostic with some atypical spindle cells in a background of necrosis. Given the concern for spinal cord compression, an MRI of the thoracic spine was performed which revealed enhancing lesions at the T3 posterior elements with epidural involvement and compression, as well as posterior T4 vertebral body. MRI of the cervical spine was without evidence of disease. Ultimately, the patient underwent a T3 laminectomy with a T2 and T4 partial laminectomy and a posterior thoracic spine fusion of T2-T5 with screws, rods, and allograft along with resection of a spinal epidural mass. Pathologic review of the biopsied epidural mass revealed an intimal sarcoma, with epithelioid and pleomorphic features, of high grade involving bone. There were 19 mitoses per 10 high-power field. Focal tumor necrosis was present (<50%). Immunohistochemistry noted rare scattered tumor cells positive for SMA, CMA (5/2), and AE (1/3), but was negative for S100, CK 7, HMB45, CK19, CK (5/6), EMA, CD31, and CD34. GMS and AFB special stains were negative for microorganisms. Additional immunostaining performed showed that the tumor cells were positive for SMA and desmin and negative for STAT-6 and SAT2B. BCL-1 stain revealed nuclear reactivity and few scattered tumor cells. INI-1 was retained. FISH was performed and demonstrated amplification of MDM2. Furthermore, CT abdomen/pelvis was negative for any metastatic disease. The patient was discharged home with pain control and instructions to follow-up outpatient with pulmonology, medical oncology, radiation oncology, and neurosurgery.

## 3. Follow-Up

The patient completed ten cycles of palliative radiation therapy to the T2-T5 region for a total dose of 3000 cGy. Following completion, he developed pleuritic chest pain and shortness of breath. He presented to a different hospital and underwent CT chest angiogram and was diagnosed with large right proximal pulmonary embolism and he was started on apixaban. He subsequently reported several episodes of blood-streaked sputum after initiation of anticoagulation. PET/CT was performed showcasing extensive hypermetabolic pulmonary nodules/masses, right much greater than left, and hypermetabolic poorly characterized right hilar and subcarinal lymphadenopathy along with concern for right pulmonary artery invasion. The patient was followed up with medical oncology and was recommended palliative chemotherapy with doxorubicin for his diagnosis of intimal sarcoma. His transthoracic echo demonstrated a normal ejection fracture of 60–65%. His treatment course was complicated by nausea, vomiting, oral mucositis, fatigue, weight loss, and insomnia. Additionally, he developed anemia and neutropenia requiring pegfilgrastim. He later developed fever and was treated with antibiotics for pneumonia. The patient completed six cycles of doxorubicin therapy. Posttreatment CT with contrast demonstrated reduction in size of pulmonary metastases and stable-appearing nonocclusive thrombus within the right main pulmonary artery (Figure 2(b)). Repeat molecular testing revealed CDK4 amplification, and he is currently receiving palbociclib. He was later admitted for shortness of breath and was newly diagnosed with systolic heart failure, likely due to doxorubicin cardiotoxicity. He received three cycles of palbociclib. Repeat CT chest with contrast showed stable disease (Figure 2(c)).

## 4. Discussion

In this case, the patient presented with vague symptoms of cough, dyspnea, and weight loss. Initial imaging modalities, including chest X-ray and CT chest with contrast, were concerning for extravascular tumor compression from primary lung carcinoma. CT angiogram revealed a filling defect suspicious of an intravascular mass, rather than extravascular tumor invasion. PET/CT scan identified pulmonary artery involvement but was unable to differentiate between intravascular or extravascular etiologies. To arrive at the final diagnosis of PAIS with metastases, several different imaging modalities, in addition to a tissue biopsy, were employed.

A multimodal approach, utilizing several imaging modalities to visualize the thorax and vasculatures via CT, PET/CT, and MRI, is the best way to rapidly diagnose PAIS [1,2]. The diagnostic challenge lies in the complexity of distinguishing: (1) whether the mass is intravascular versus extravascular, (2) whether this mass is malignant or benign, and (3) if the mass is intravascular, whether it is a tumor thrombus, tumor mass, or pseudoaneurysm. Other reported cases suggest that the diagnostic process is often convoluted with difficulties in establishing a diagnosis and subsequent delay in initiating appropriate therapy. To differentiate an intravascular mass from extravascular mass, a CT angiogram would be necessary to directly visualize the pulmonary vasculatures compared to a CT chest with contrast. On CT chest, PAIS is characterized by multiple pulmonary nodules and hilar or mediastinal adenopathy, in comparison to pulmonary thromboembolisms, which often have associated wedge-shaped infarcts [2, 3]. The “wall eclipsing sign,” where the tumor eclipses one or both walls of the main pulmonary artery or its branches, is pathognomonic for PAIS on CT angiography [4]. In contrast, pulmonary thromboembolism is in the shape of a saddle [2, 4]. Furthermore, high uptake on PET would suggest a malignancy, whereas low uptake would suggest a nonmalignant etiology such as pulmonary thomboembolism [2, 5]. Prior case reports utilized and integrated the findings of all three modalities in order to rapidly diagnose PAIS [4, 6]. Cardiac MRI can be utilized to discern neoplastic from thrombotic components by allowing proper visualization of the vasculature and tissue edema [7]. In addition, PAIS demonstrates diffusion restriction and heterogeneous enhancement, in comparison to pulmonary thromboembolism [8].

Tissue biopsy by endobronchial ultrasound-guided transbronchial needle aspiration followed by immunohistochemistry remains gold standard for diagnosis [5]. In this case, the endobronchial biopsy was unrevealing, but a biopsy of the metastatic mass confirmed the diagnosis. Establishing a definitive diagnosis is essential as treatment and prognosis vary drastically for PAIS, pulmonary thromboembolism, and other mimics such as lung carcinoma. It is interesting as this patient was diagnosed with both PAIS and later pulmonary embolism.

There is no standard treatment to date, and management often includes a multidisciplinary approach involving surgery, radiation, chemotherapy, and targeted therapy. Surgery is considered first-line treatment in order to relieve pulmonary artery obstruction and associated pulmonary hypertension and to palliate symptoms [9]. Surgical intervention is dependent on the location on the extension of the mass and can include pulmonary endarterectomy, pneumonectomy, or a lobectomy. Additionally, surgery is noncurative as it is difficult to achieve clear margins; therefore, adjuvant chemotherapy and radiation are utilized, in addition to surgery, to achieve better disease control [9, 10]. For systemic therapy, anthracyclines are most commonly used along with taxanes, ifosfamide, platinum, and gemcitabine [9, 10]. Despite the above treatment options, prognosis remains poor due to the difficulty in achieving complete disease control. In the metastatic setting, chemotherapy and radiation therapy should be considered prior to any surgical intervention [9]. In the presented case, given the high-grade nature of PAIS with pulmonary and bone metastasis, palliative chemotherapy with doxorubicin was considered. After six cycles of doxorubicin, the patient had a partial response to his disease; hence, palbociclib (CDK4/6 inhibitor) was considered. Among several other alterations, CDK4 amplification was found to be prominent in intimal sarcomas [11]. The role of palbociclib treatment of intimal sarcomas with CDK4 amplification is still under investigation and remains unclear.

## 5. Conclusion

PAIS is a rare entity often resembling lung carcinoma or pulmonary thromboembolism as a result of nonspecific symptomatology and analogous imaging findings, thereby delaying diagnosis and treatment. There is no standard of treatment for PAIS and its treatment often involves a multidisciplinary approach, with anthracyclines as the main systemic agent.

## Data Availability

No data were used to support this study.
